# Four new species of the trapdoor spider genus *Conothele* Thorell, 1878 (Araneae, Halonoproctidae) from China

**DOI:** 10.3897/zookeys.833.32736

**Published:** 2019-04-01

**Authors:** Hao Liu, Xin Xu, Zengtao Zhang, Fengxing Liu, Daiqin Li

**Affiliations:** 1 State Key Laboratory of Biocatalysis and Enzyme Engineering, Centre for Behavioural Ecology and Evolution (CBEE), School of Life Sciences, Hubei University, 368 Youyi Road, Wuhan 430062, Hubei Province, China Hubei University Wuhan China; 2 College of Life Sciences, Hunan Normal University, 36 Lushan Road, Changsha 410081, Hunan Province, China Hunan Normal University Changsha China; 3 Department of Biological Sciences, National University of Singapore, 14 Science Drive 4, 117543, Singapore National University of Singapore Singapore Singapore

**Keywords:** Araneae, China, COI, DNA barcode, Mygalomorphae, taxonomy

## Abstract

Herein four species of the trapdoor spider genus *Conothele* Thorell, 1878 collected from China are described as new to science based on the female genital morphology: *C.baisha***sp. n.** (Hainan Province), *C.baoting***sp. n.** (Hainan Province), *C.linzhi***sp. n.** (Tibet), and *C.jinggangshan***sp. n.** (Jiangxi Province). For two Hainan species, *C.baisha***sp. n.** and *C.baoting***sp. n.**, between which it is difficult to distinguish solely based on female genital morphology, additional diagnoses derived from species-specific nucleotide substitution information and genetic distances using the mitochondrial gene, cytochrome c oxidase subunit I are provided.

## Introduction

**Figure 1.**
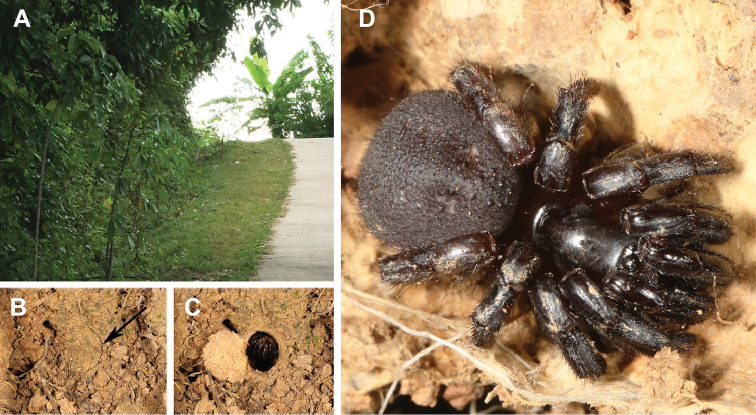
Microhabitat, burrow with a trapdoor, and general somatic morphology of *Conothelebaisha* sp. n. **A** microhabitat **B, C** burrow exterior **B** the trapdoor with the door closed **C** the trapdoor with door open **D** female (LH-2017-089; Jishi Village, Changjiang County, Hainan Province, China).

*Conothele* Thorell, 1878 is a genus of trapdoor spiders belonging to the family Halonoproctidae Pocock, 1901 (Opisthothelae: Mygalomorphae) that was recently elevated from the family Ctenizidae based on molecular-based evidence (Godwin et al. 2018). Like many poor-dispersal, ground-dwelling trapdoor spiders (although some species of *Ummidia* Thorell, 1875 disperse by ballooning (Coyle 1985; Fisher et al. 2014)), *Conothele* spiders construct underground burrows which are lined with silk and opened to the surface with a trapdoor. The trapdoor is usually covered with a layer of soil, leaf litter, and/or moss, which blend well in the surrounding environment, making them difficult to locate in nature (Fig. 1; Bond and Coyle 1995; Xu et al. 2017a; Yang and Xu 2018).

*Conothele* was previously placed in the family Ctenizidae. However, the two Ctenizidae subfamilies, Ctenizinae and Ummidiinae (Raven 1985; Ortiz 2007), and even the entire family were not monophyletic (Hedin and Bond 2006; Ayoub et al. 2007; Bond et al. 2012; Opatova et al. 2013). Recently, Godwin et al. (2018) re-limited the whole family and subfamilies based on molecular phylogenetic evidence, and split it into two families, Halonoproctidae and Ctenizidae. Halonoproctidae now comprises six genera and 87 species belonging to two subfamilies, Ummidiinae Ortiz, 2007 (*Conothele*, *Latouchia* Pocock, 1901, and *Ummidia*) and Halonoproctinae Pocock 1901 (*Bothriocyrtum* Simon, 1891, *Cyclocosmia* Ausserer, 1871, and *Hebestatis* Simon, 1903) (Godwin et al. 2018; World Spider Catalog 2019).

The two ummidiin genera *Conothele* and *Ummidia* share some common morphological and behavioral characters, thus they were considered as undistinguishable (Main 1985; Decae 2010). One of the most obvious shared features by two genera is the presence of a saddle depression on tibia III (Gertsch 1979; Coyle 1981; Ortiz 2007), leading some authors to consider both genera as synonyms (Decae 2010). However, these two genera are completely separated geographically, with *Conothele* being distributed in the Orient and Australasian regions, and *Ummidia* being found in the New World and Mediterranean regions (Xu et al. 2017a; Godwin et al. 2018; Yang and Xu 2018). In addition, they are reciprocally monophyletic, and currently considered as valid genera based on phylogenetic analyses (Godwin et al. 2018).

*Conothele* contains 26 described species that are widely distributed in the Orient (China, India, Japan, Laos, Myanmar, Sumatra) and Australasia (World Spider Catalog 2019). Until now, only seven species have been described from China primarily based on either female or male morphology (World Spider Catalog 2019), including *C.taiwanensis* (Tso, Haupt & Zhu, 2003) (♂♀; Taiwan Province), *C.baiyunensis* (Xu, Xu & Liu, 2017) (♀; Guangzhou Province), *C.daxinensis* (Xu, Xu & Li, 2017) (♀; Guangxi Province), *C.sidiechongensis* (Xu, Xu & Liu, 2017) (♀; Yunnan Province), *C.yundingensis* (Xu, Xu & Li, 2017) (♀; Yunnan Province), *C.cangshan* (Yang & Xu, 2018) (♂; Yunnan Province) and *C.deqin* (Yang & Xu, 2018) (♂; Yunnan Province).

**Figure 2.**
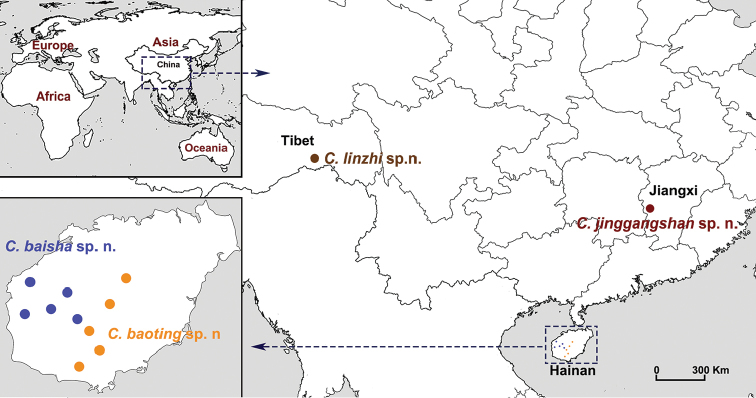
A map showing the distribution of four new species.

In this study, we diagnosed and described four new *Conothele* species collected in China based on female morphology as we were unable to obtain adult males (Fig. 2). As in other halonoproctid studies (Xu et al. 2017a; Yang and Xu 2018), both male and female morphology should be described for a new species; however, often it is impractical or impossible to collect adult males by direct searching or by excavating burrows. The standard DNA alignment of the mitochondrial cytochrome *c* oxidase subunit (COI), which provides the species-specific nucleotide substitution information in the animal barcoding gene region, has been widely used to diagnose species (Brower 2010; Cook et al. 2010; Planas and Ribera 2015; Xu et al. 2015, 2017b). Therefore, for the two new species from Hainan Province (*Conothelebaisha* sp. n. and *C.baoting* sp. n.) that show similar morphology and considerable intraspecific variations in female genitalia, we provided additional evidence of species-specific nucleotide substitutions and genetic distances based on COI to support our identifications and for future verification of males.

## Materials and methods

All specimens were collected from Tibet, Hainan, Jiangxi Provinces, China (Fig. 2). The right four legs of adult females were removed and stored in 100% ethanol at -80 °C for the molecular work. The rest of each specimen was stored as a voucher in 75–80% ethanol for morphological examination. All the voucher specimens were examined under an Olympus SZX16 stereomicroscope, and they were photographed using a Leica M205C digital microscope. Genitalia were cleaned by Protease K digest for 3 hrs at 56 °C. All the voucher specimens were deposited at the **CBEE**(Centre for Behavioural Ecology and Evolution), School of Life Sciences, Hubei University, Wuhan, China. All measurements were carried out under a Leica M205C digital microscope and given in millimeters. Standard measurements were made following Decae (2010). Measurements of legs and palps are given in the following order: Leg total length (femur + patella + tibia + metatarsus + tarsus), palp total length (femur + patella + tibia + tarsus).

Abbreviations used are:

**ALE** anterior lateral eye;

**AME** anterior median eye;

**PLE** posterior lateral eye;

**PME** posterior median eye;

**MOA** median ocular area;

**PMS** posterior median spinneret;

**PLS** posterior lateral spinneret;

**TL** total length (including chelicerae but excluding spinnerets).

We extracted the total genomic DNA using the universal genomic DNA extraction kit (CWBIO) from one or two right legs per specimen depending on the size of the legs. The 25 μL PCR reaction included 12.5 µl 2 × TaqMaster Mix (TIANGEN), 9.5 µl double-distilled H_2_O (ddH_2_O), 1 µl genomic DNA and 1 µl of each forward and reverse primer (10 µM). The primer pairs of COI were LCO1490 (5’-GGTCAACAAATCATAAAGATATTGG-3’) and HCO2198 (5’-TAAACTTCAGG GTGACCAAA AAATCA-3’) (Folmer et al. 1994). The PCR reaction protocol: initial denaturation at 94 °C for 5 min; 35 cycles of denaturation at 94 °C for 30 s, annealing at 40 °C for 45s and elongation at 72 °C for 1 min, and final extension at 72 °C for 10 min. The PCR products were visualized by agarose gel electrophoresis (1% agarose). All PCR products were purified and sequenced at the TSINGKE Biological Technology (Wuhan China) or Sunny Biological (Shanghai China). The species-specific nucleotide substitutions in the standard DNA barcode alignment and genetic distances were identified using MEGA v6 (Tamura et al. 2013; Xu et al. 2017b).

## Taxonomy

### Genus Conothele

Taxon classificationAnimaliaAraneaeHalonoproctidae

Thorell, 1878

#### Type.

*Conothelemalayana* (Doleschall 1859): 5, pl. 5, fig. 8 (described female).

#### Diagnosis.

The genus *Conothele* can be distinguished from all other Halonoproctidae genera other than *Ummidia* by the presence of a saddle depression on tibia III (Coyle 1981; Ortiz 2007; Decae 2010). *Conothele* differs from *Ummidia* by their burrowing habits. The former constructs a short, parallel to the surface of ground, superficial burrow, whereas the latter digs a several centimeters long burrow in the soil (Haupt 2006). Moreover, the geographical ranges of *Ummidia* and *Conothele* are completely separated, with *Conothele* being distributed in the Orient and Australasian regions, and with *Ummidia* being distributed in the New World and Mediterranean regions (Xu et al. 2017a; Godwin et al. 2018; Yang and Xu 2018; World Spider Catalog 2019).

### Conothele linzhi sp. n.

Taxon classificationAnimaliaAraneaeHalonoproctidae

http://zoobank.org/93117D0B-1A52-4CC3-9E67-044B44BB7DAF

Fig. 3

#### Holotype.

Female (LH-2017-051), collected in Baishuwang Garden Roadside, Bayi Town, Linzhi City, Tibet, China, 29.6106N, 94.4040E, 2980 m a.s.l., 14 July 2017, collected by FX Liu, ZT Zhang, J Chen and J Liu (CBEE).

#### Paratypes.

3 females (LH-2017-046, LH-2017-048, LH-2017-050), collected at the same locality as the holotype (CBEE).

#### Diagnosis.

**Figure 3.**
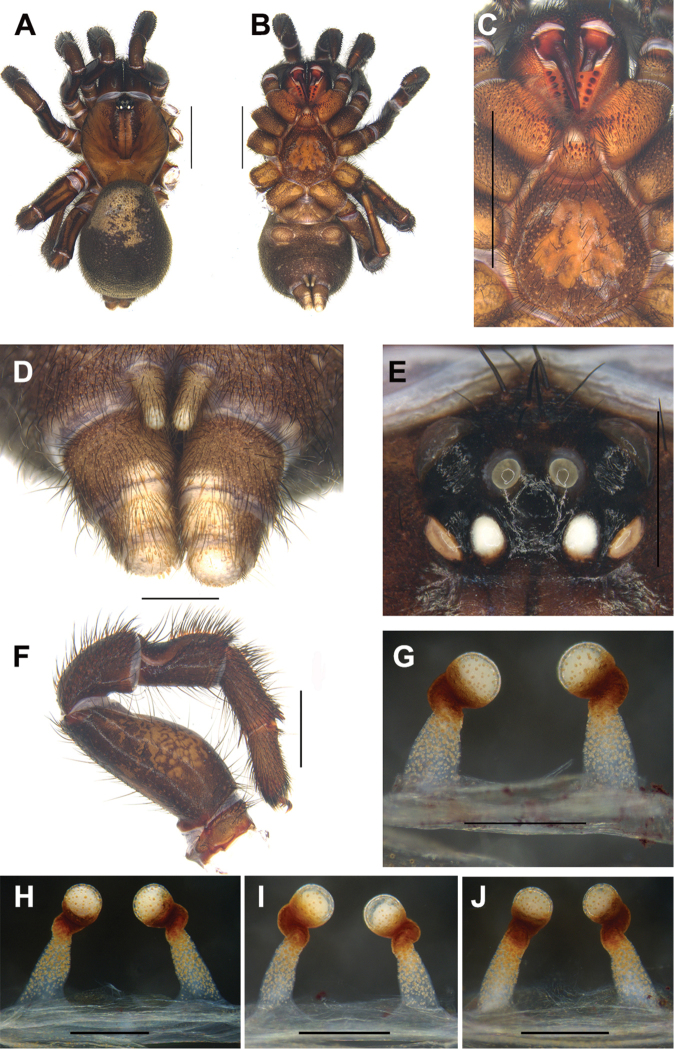
General somatic morphology and female genitalia of *Conothelelinzhi* sp. n. **A–G** holotype (LH-2017-051) **A** dorsal view **B** ventral view **C** chelicerae, labium, coxae of palp and sternum, ventral view **D** spinnerets, ventral view **E** eyes, dorsal view **F** left leg III, prolateral view **G–J** female genitalia, dorsal view **H–J** paratypes **H** (LH-2017-046) **I** (LH-2017-048) **J** (LH-2017-050). Scale bars: 5 mm (**A–C**); 1 mm (**D, E**); 2 mm (**F**); 0.5 mm (**G–J**).

Females of *C.linzhi* sp. n. can be distinguished from those of the other *Conothele* species by an obviously large irregularly duct-like sigillum in the sternum center (Fig. 3C); by the terminal lobes of spermathecae hemisphere-shaped; by the distal part of stalks Z-shaped and tilted slightly anteriorly (Fig. 3G-J).

#### Description.

TL 19.26; chelicerae length 2.51, carapace 7.39 long, 7.15 wide; opisthosoma 9.43 long, 7.38 wide. Carapace brownish black, glabrous, with a few slender setae on or behind the eye tubercle. Caput arched. Fovea deep and darker (Fig. 3A). Eight eyes in two rows, with the anterior eye row procurved, and the posterior eye row straight (Fig. 3E); eye group 0.93 long, 1.48 wide; ALE-AME 0.29, AME-AME 0.18, PLE-PME 0.08, PME-PME 0.38; MOA 0.64 long, front width 0.56, back width 0.82; ALE: AME: PLE: PME (0.48: 0.19: 0.33: 0.21). Many slender setae on clypeus (Fig. 3E). Chelicerae black (dorsal view); inner margin with 6 teeth, outer margin with 9 teeth. Labium, coxae of palp and sternum brownish black (Fig. 3C). Labium 1.27 long, 1.47 wide, with 19 conspicuous cuspules. Coxae of palp 2.78 long, 1.86 wide, with approx. 68 conspicuous cuspules (the right one, ventral view) (Fig. 3C). Sternum 4.60 long, 4.65 wide, with an obviously large, irregularly shaped sigillum in the center and with many setae (Fig. 3C).

Legs brownish black, with long and short black dense setae. Tibia III with a saddle-like depression dorsally on the basal part, and the depression is smaller than that of the other *Conothele* species (Fig. 3F). Palp with a single tarsal claw and a denticle on the claw. Legs each with three tarsal claws, paired claws with one denticle. Leg formula: IV, I, II, III. Measurements of palp and legs: palp 13.25 (4.99+2.02+3.51+2.73), leg I 15.25 (5.65+2.66+3.61+2.12+1.21), leg II 12.74 (4.52+2.13+2.65+2.06+1.38), leg III 12.70 (4.52+1.84+2.33+2.00+2.01), leg IV 16.22 (5.22+2.24+2.77+3.13+2.86).

Opisthosoma ellipsoid and black, scattered with thick and slender black setae. Spinnerets brown (Fig. 3D), PMS one-segmented, 0.86 long, PMS-PMS 0.23; PLS divided into three sections, 2.17 long. Female genitalia with a pair of spermathecae slightly tilted to the middle; the terminal lobes of spermathecae hemisphere-shaped; stalks sclerotized and Z-shaped distally, and tilted slightly anteriorly (Fig. 3G-J).

#### Male.

Unknown.

#### Etymology.

The species epithet, a noun in apposition, refers to the type locality.

#### Distribution.

Tibet (Linzhi City).

### Conothele jinggangshan sp. n.

Taxon classificationAnimaliaAraneaeHalonoproctidae

http://zoobank.org/B259FF23-9B58-40C0-8F4B-11905347CDA9

Fig. 4

#### Holotype.

Female (LH-2017-225), collected in Revolutionary Martyrs Cemetery, Ciping Town, Jinggangshan City, Jian City, Jiangxi Province, China, 26.5881N, 114.1599E, 910 m a.s.l., 12 September 2017, collected by FX Liu, F Li (CBEE).

#### Diagnosis.

**Figure 4.**
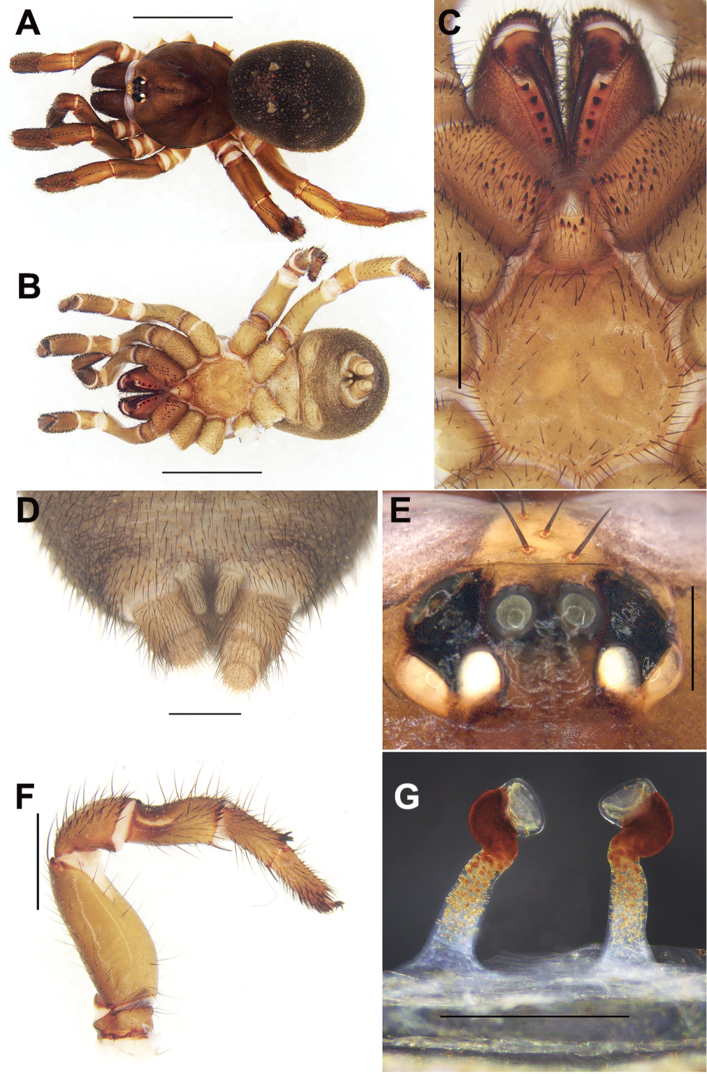
General somatic morphology and female genitalia of *Conothelejinggangshan* sp. n. (holotype, LH-2017-225) **A** dorsal view **B** ventral view **C** chelicerae, labium, coxae of palp and sternum, ventral view **D** spinnerets, ventral view **E** eyes, dorsal view **F** left leg III, prolateral view **G** female genitalia, dorsal view. Scale bars: 5 mm (**A, B**); 1 mm (**D**); 2 mm (**C, F**); 0.5 mm (**E, G**).

Female of *C.jinggangshan* sp. n. can be distinguished from those of the other *Conothele* species by the sternum with a pair of obvious elliptic sigilla (Fig. 4C); by the distal part of stalks which are outwardly and then inwardly bend, somewhat semi-circle-like (Fig. 4G).

#### Description.

TL 13.74; chelicerae length 1.77, carapace 6.62 long, 5.40 wide; opisthosoma 6.89 long, 5.70 wide. Carapace dark brown, glabrous, with a few slender setae on or behind the eye tubercle (Fig. 4A). Caput arched. Fovea deep and dark (Fig. 4A). Eye tubercle black. Eight eyes in two rows, with the anterior eye row procurved, and the posterior eye row slightly recurved (Fig. 4E); eye group 0.73 long, 1.38 wide; ALE-AME 0.20, AME-AME 0.14, PLE-PME 0.02, PME-PME 0.46; MOA 0.51 long, front width 0.48, back width 0.86; ALE: AME: PLE: PME (0.34: 0.17: 0.30: 0.19). Four slender setae on clypeus (Fig. 4E). Chelicerae dark brown (dorsal view); inner margin with five teeth, outer margin with seven teeth. Labium, coxae of palp and sternum brown (Fig. 4C). Labium 0.82 long, 1.00 wide, with four conspicuous cuspules. Coxae of palp 2.03 long, 1.31 wide, with approx. 41 conspicuous cuspules (the right one, ventral view) (Fig. 4C). Sternum 3.09 long, 2.74 wide, with a pair of obvious elliptic sigilla and with small number of setae (Fig. 4C).

Legs brown, light brown ventrally, with long and short brown sparse setae. Basal part of tibia III with saddle-like depression dorsally (Fig. 4F). Palp with a single tarsal claw and with two denticles on the claw. Legs each with three tarsal claws, paired claws with one denticle. Leg formula: IV, I, II, III. Measurements of palp and legs: palp 9.07 (3.40+1.42+2.27+1.98), leg I 9.89 (3.65+1.93+2.41+1.04+0.86), leg II 9.40 (3.21+1.66+2.13+1.23+1.17), leg III 9.35 (3.20+1.29+2.01+1.36+1.49), leg IV12.09 (4.14+1.74+2.50+2.16+1.55).

Opisthosoma ellipsoid, black, scattered with slender short black setae. Spinnerets brown (Fig. 4D), PMS short and one-segmented, 0.64 long, PMS-PMS 0.19; PLS divided into three sections, 1.38 long. Genitalia with a pair of spermathecae, each stalk slender, long, distally sclerotized and folded, which is first bent outwards and then inwards, semi-circle-like; with bowl-shaped lobes (Fig. 4G).

#### Male.

Unknown.

#### Etymology.

The species epithet, a noun in apposition, refers to the type locality.

#### Distribution.

Jiangxi Province (Jinggangshan City).

### Conothele baisha sp. n.

Taxon classificationAnimaliaAraneaeHalonoproctidae

http://zoobank.org/464B6E9B-B04A-49F8-8516-AE79A33A12A7

Figs 5 , 6

#### Holotype.

Female (LH-2017-136), collected in Nanmeiling, Yacha Town, Baisha County, Hainan Province, China, 19.1075N, 109.4227E, 250 m a.s.l., 10 August 2017, collected by FX Liu, D Li, ZT Zhang, X Xu (CBEE).

#### Paratypes.

2 females (LH-2017-128, LH-2017-135) collected at the same locality as the holotype (CBEE); 1 female (LH-2017-080), collected in Yalong Village, Tianan Township, Donghe Town, Dongfang City, Hainan Province, China, 18.9947N, 108.8976E, 170 m a.s.l., 5 August 2017; 1 female (LH-2017-089), collected in Jishi Village, Changjiang County, Hainan Province, China, 19.2305N, 109.0730E, 170 m a.s.l., 6 August 2017; 1 female (LH-2017-090), collected in Bawangling National Forest Park, Baoshan village, Changjiang County, Hainan Province, China, 19.0757N, 109.0822E, 210 m a.s.l., 7 August 2017; 1 female (LH-2017-161), collected in Shiyixinyi Village, Wuzhishan City, Hainan Province, China. 18.9122N, 109.5118E, 290 m a.s.l., 11 August 2017, all collected by FX Liu, D Li, ZT Zhang, X Xu (CBEE).

#### Diagnosis.

**Figure 5.**
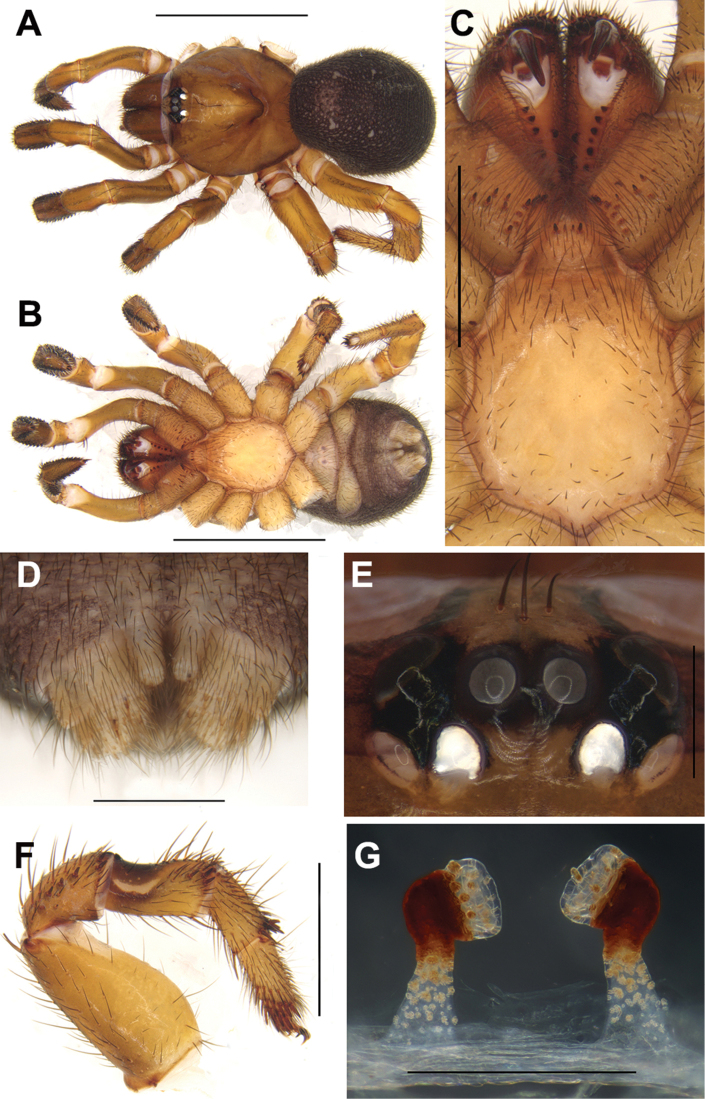
General somatic morphology and female genitalia of *Conothelebaisha* sp. n. holotype (LH-2017-136) **A** dorsal view **B** ventral view **C** chelicerae, labium, coxae of palp and sternum, ventral view **D** spinnerets, ventral view **E** eyes, dorsal view **F** left leg III, prolateral view **G** female genitalia, dorsal view. Scale bars: 5 mm (**A, B**); 1 mm (**D**); 2 mm (**C, F**); 0.5 mm (**E, G**).

Female genitalia of *C.baisha* sp. n. resembles *C.daxinensis* (Xu, Xu & Li, 2017), but can be distinguished from the latter by the spermathecae with each stalk sturdy, short, simple and direct (Fig. 5G). It can be also distinguished from *C.baoting* sp. n. by short stalks without the trench between the distal part of the stalks and the lobes. Moreover, *C.baisha* sp. n. can be distinguished from *C.baoting* sp. n. by the following unique nucleotide substitutions in the standard DNA barcode alignment: A (13), G (97), A (134), T (157), A (172), G (196), C (205), A (223), T (224), A (253), G (280), C (302), G (304), C (322), A (421), G (424), A (502), G (520), A (592), A (634), G (637).

#### Description.

TL10.35; chelicerae length 1.49, carapace 4.76 long, 4.28 wide; opisthosoma 4.83 long, 4.22 wide. Carapace brown, glabrous, with a few slender setae on or behind the eye tubercle (Fig. 5A). Caput arched. Fovea deep and brown (Fig. 5A). Eye tubercle black. Eight eyes in two rows, with the anterior eye row slightly procurved, and the posterior eye row slightly recurved (Fig. 5E); eye group 0.56 long, 1.18 wide; ALE-AME 0.14, AME-AME 0.10, PLE-PME 0.04, PME-PME 0.36; MOA 0.43 long, front width 0.43, back width 0.70; ALE: AME: PLE: PME (0.34: 0.19: 0.23: 0.16). Three slightly thick setae on clypeus (Fig. 5E). Chelicerae dark brown (dorsal view); inner margin with three teeth, outer margin with seven teeth. Labium, coxae of palp and sternum brown (Fig. 5C). Labium 0.53 long, 0.80 wide, with four conspicuous cuspules. Coxae of palp 1.50 long, 1.10 wide, with approx. 13 conspicuous cuspules (the right one, ventral view) (Fig. 5C). Sternum 2.97 long, 2.35 wide, with a large smooth area which lacks setae in the center, but many setae outside this area (Fig. 5C).

Legs brown, with long and short brown sparse setae. Basal part of tibia III with saddle-like depression dorsally (Fig. 5F). Palp with a single tarsal claw, with two denticles on the claw. Legs each with three tarsal claws, paired claws with one denticle. Leg formula: IV, I, III, II. Measurements: palp 6.86 (2.63+1.25+1.42+1.56), leg I 8.11 (2.97+1.46+1.83+0.99+0.86), leg II 7.18 (2.55+1.36+1.54+0.84+0.89), leg III 7.46 (2.92+0.92+1.45+0.92+1.25), leg IV 8.64 (3.07+1.22+1.62+1.42+1.31). Leg II and leg III are almost the same length.

Opisthosoma ellipsoid and black, scattered with many slender, short black setae. Spinnerets brown (Fig. 5D), PMS short and one-segmented, 0.48 long, PMS-PMS 0.08; PLS divided into three sections, 0.78 long. Genitalia with a pair of spermathecae, terminating with face-to-face bowl-shaped lobes; stalks sclerotized distally, each stalk sturdy, short, simple and direct, without the trench between the distal part of the stalks and the lobes (Fig. 5G).

#### Variation.

**Figure 6.**
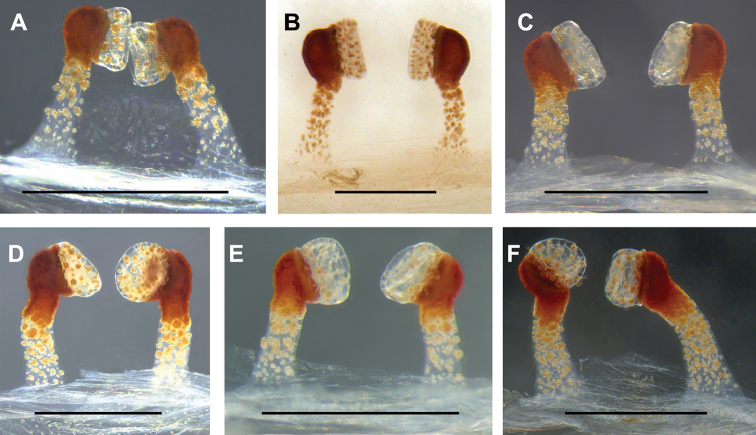
Female genitalia of the paratypes of *Conothelebaisha* sp. n. showing the intraspecific variations in spermathecae. **A** (LH-2017-080) **B** (LH-2017-089) **C** (LH-2017-090) **D** (LH-2017-128) **E** (LH-2017-135) **F** (LH-2017-161), dorsal view. Scale bars: 0.5 mm.

The female genitalia show considerable intraspecific variations: the spermathecae stalks of the holotype (Fig. 5G) and some paratypes are unbent (Fig. 6A–C, E), or slightly curved (Fig. 6D), or the stalk on the left is tilted to the right by ca. 30°, and the right stalk is curled distally (Fig. 6F). The spermathecae of all samples are face to face, except for one (Fig. 6F).

#### Male.

Unknown.

#### Etymology.

The species epithet, a noun in apposition, refers to the type locality.

#### Distribution.

Hainan Province (Baisha County, Changjiang County, Dongfang City, Wuzhishan City).

#### GenBank accession numbers.

LH-2017-080: MK454955; LH-2017-089: MK454956; LH-2017-090: MK454957; LH-2017-128: MK454958; LH-2017-135: MK454959; LH-2017-136: MK454960; LH-2017-161: MK454961.

#### Remarks.

The mean intraspecific genetic distance of *C.baisha* sp. n. is 1.25 % and 1.23 % using Kimura 2-parameter (K2P) model and *p*-distance model, respectively. The interspecific genetic distance between *C.baisha* sp. n. and *C.baoting* sp. n. is 5.78 % and 5.49 % using K2P and *p*-distance, respectively. This interspecific genetic distance in *Conothele* is comparable to other mygalomorphs identified at 5–6% (Hamilton et al. 2011, 2014).

### Conothele baoting sp. n.

Taxon classificationAnimaliaAraneaeHalonoproctidae

http://zoobank.org/BB49CB7D-E6A6-4994-A5F3-3F63720DFFA5

Figs 7 , 8

#### Holotype.

Female (LH-2017-205), collected in Maoding Village, Shiling Town, Baoting County, Hainan Province, China, 18.6987N, 109.7563E, 160 m a.s.l., 20 August 2017, collected by FX Liu, D Li, X Xu (CBEE).

#### Paratypes.

1 female (LH-2017-209), collected at the same locality as the holotype (CBEE); 5 females (LH-2017-165, LH-2017-166, LH-2017-167, LH-2017-168, LH-2017-169), collected in Wuzhishan City, Hainan Province, China, 18.8147N, 109.5124E, 260–470 m a.s.l., 12 August 2017, collected by FX Liu, D Li, ZT Zhang, X Xu (CBEE); 4 females (LH-2017-179, LH-2017-180, LH-2017-196, LH-2017-198), collected in Qiongzhong County, Hainan Province, China, 18.9899N, 109.6720E, 190–380 m a.s.l., 14–17 August 2017, collected by FX Liu, D Li, X Xu (CBEE); 1 female (LH-2017-187), collected in Wupo Town, Tunchang County, Hainan Province, China, 19.1380N, 110.0625E, 90 m a.s.l., 15 August 2017, collected by FX Liu, D Li, X Xu (CBEE); 3 females (LH-2017-211, LH-2017-212, LH-2017-213), collected in Baoqian Village, Tianya District, Sanya Ciy, Hainan Province, China, 18.3931N, 109.4224E, 90 m a.s.l., 22 August 2017, collected by FX Liu, D Li, X Xu (CBEE).

#### Diagnosis.

**Figure 7.**
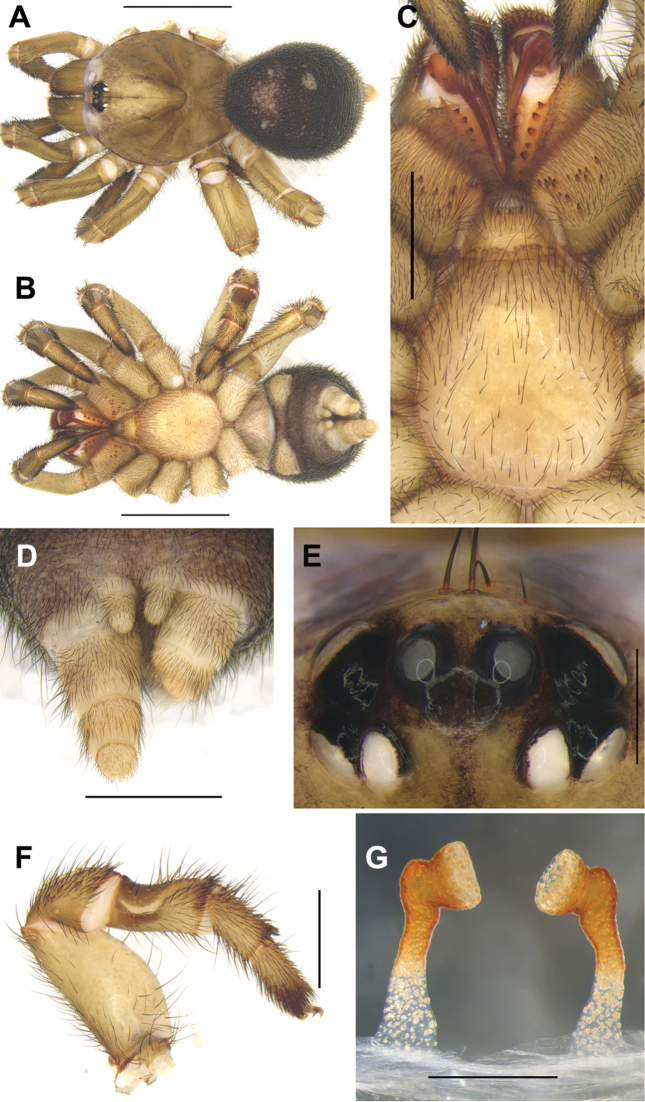
General somatic morphology and female genitalia of *Conothelebaoting* sp. n. holotype (LH-2017-205) **A** dorsal view **B** ventral view **C** chelicerae, labium, coxae of palp and sternum, ventral view **D** spinnerets, ventral view **E** eyes, dorsal view **F** left leg III, prolateral view **G** female genitalia, dorsal view. Scale bars: 5 mm (**A, B**); 2 mm (**C, D, F**); 0.5 mm (**E, G**).

Females of *C.baoting* sp. n. can be distinguished from those of other *Conothele* species by the spermathecae with plate-shaped lobes, each stalk slender, long, distally sclerotized and thickened, and narrowest in the middle. It can be distinguished from *C.baisha* sp. n. by long stalks each with an obvious trench between the distal part of the stalk and the lobe (Fig. 7G). Moreover, *C.baoting* sp. n. can be diagnosed from *C.baisha* sp. n. by the following unique nucleotide substitutions in the standard DNA barcode alignment: G (13), T (97), G (134), A (157), G (172), A (196), T (205), G (223), C (224), T (253), A (280), T (302), A (304), T (322), C (376), G (421), A (424), G (502), A (520), G (592), G (634), A (637).

#### Description.

TL 14.71, chelicerae length 1.80, carapace 6.92 long, 6.20 wide; opisthosoma 6.52 long, 5.40 wide. Carapace light brown, glabrous, with a few slender setae on or behind the eye tubercle (Fig. 7A). Caput arched. Fovea deep and brown (Fig. 7A). Eye tubercle black. Eight eyes in two rows, with both two eye rows straight (Fig. 7E); eye group 0.76 long, 1.39 wide; ALE-AME 0.19, AME-AME 0.25, PLE-PME 0.03, PME-PME 0.57; MOA 0.66 long, front width 0.56, back width 0.90; ALE: AME: PLE: PME (0.38: 0.14: 0.32: 0.15). Three slightly thick setae on clypeus (Fig. 7E). Chelicerae light brown (dorsal view); inner margin with five teeth, outer margin with seven teeth. Labium, coxae of palp and sternum brown (Fig. 7C). Labium 0.84 long, 1.41 wide, with three conspicuous cuspules. Coxae of palp 2.04 long, 1.54 wide, with approx. 18 conspicuous cuspules (the right one, ventral view) (Fig. 7C). Sternum 3.91 long, 3.23 wide, with a large smooth area which has a few setae in the center and many setae outside (Fig. 7C).

Legs brown, with long and short brown dense setae. Basal part of tibia III with saddle-like depression dorsally (Fig. 7F). Palp with a single tarsal claw, with two denticles on the claw. Legs each with three tarsal claws, paired claws with two denticles. Leg formula: IV, I, II, III. Measurements: palp 8.73 (3.20+1.58+2.03+1.92), leg I 11.15 (3.99+2.17+2.49+1.49+1.01), leg II 10.37 (3.58+2.11+2.20+1.22+1.26), leg III 9.81 (3.26+1.41+2.10+1.40+1.64), leg IV 11.94 (4.13+1.72+2.25+2.17+1.67).

Opisthosoma ellipsoid, black, scattered with sparse slender, short black setae. Spinnerets brown (Fig. 7D). PMS one-segmented and short, slightly thick, 0.62 long, PMS-PMS 0.12; PLS divided into three sections, 2.23 long. Genitalia with a pair of spermathecae; spermathecae with plate-shaped lobes, each stalk slender, long, distally sclerotized and thickened, and narrowest in the middle. There is an obvious trench between the distal part of the stalk and lobe (Fig. 7G).

#### Variation.

**Figure 8.**
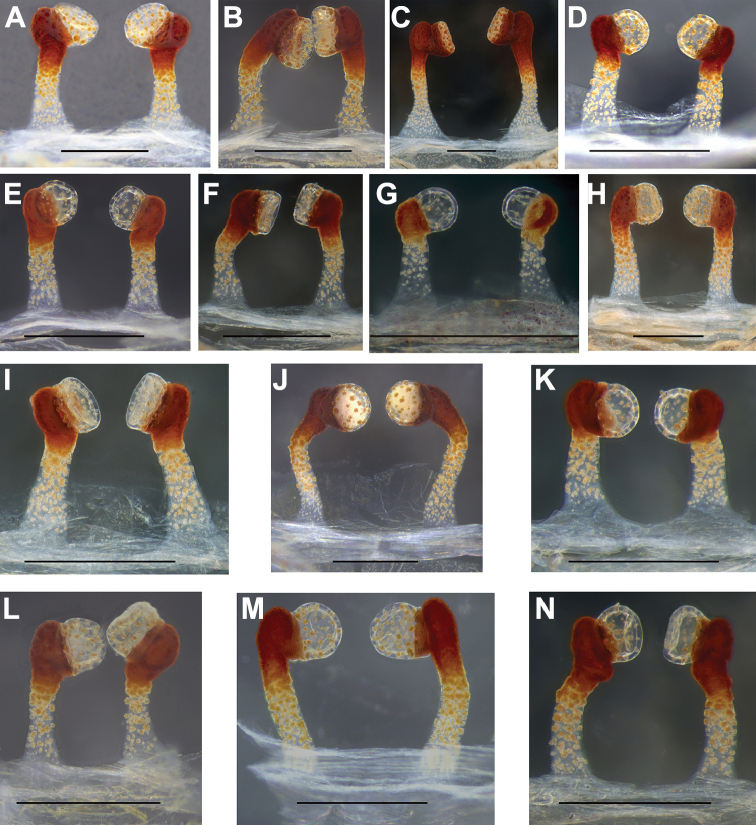
Female genitalia of the paratypes of *Conothelebaoting* sp. n. showing the intraspecific variations in spermathecae **A** (LH-2017-165) **B** (LH-2017-166) **C** (LH-2017-167) **D** (LH-2017-168) **E** (LH-2017-169) **F** (LH-2017-179) **G** (LH-2017-180) **H** (LH-2017-187) **I** (LH-2017-196) **J** (LH-2017-198) **K** (LH-2017-209) **L** (LH-2017-211) **M** (LH-2017-212) **N** (LH-2017-213) dorsal view. Scale bars: 0.5 mm.

The female genitalia show considerable intraspecific variations: the stalks of some specimens are unbent (Fig. 8A, D, E, G, H, I, K, L), while others are slightly curved (Fig. 8B, C, F, J, M, N); there are three different shapes of lobes of spermathecae, slightly globular (Fig. 8D, E, G, J, K, M, N), bowl-shaped (Fig. 8A, B, F, H, I, L), and plate-shaped (Figs 7G, 8C).

#### Male.

Unknown.

#### Etymology.

The species epithet, a noun in apposition, refers to the type locality.

#### Distribution.

Hainan Province (Baoting County, Qiongzhong County, Sanya Ciy, Tunchang County, Wuzhishan City).

#### GenBank accession numbers.

LH-2017-165: MK454962; LH-2017-166: MK454963; LH-2017-167: MK454964; LH-2017-168: MK454965; LH-2017-169: MK454966; LH-2017-179: MK454967; LH-2017-180: MK454968; LH-2017-187: MK454969; LH-2017-196: MK454970; LH-2017-198: MK454971; LH-2017-205: MK454972; LH-2017-209: MK454973; LH-2017-211: MK454974; LH-2017-212: MK454975; LH-2017-213: MK454976.

#### Remarks.

The mean intraspecific genetic distance of *C.baoting* sp. n. is 0.77 % in K2P and 0.76 % in *p*-distance.

## Supplementary Material

XML Treatment for Conothele

XML Treatment for Conothele linzhi

XML Treatment for Conothele jinggangshan

XML Treatment for Conothele baisha

XML Treatment for Conothele baoting
